# Pool Testing as a Strategy for Prevention of SARS-CoV-2 Outbreaks in Schools: Protocol for a Feasibility Study

**DOI:** 10.2196/28673

**Published:** 2021-05-28

**Authors:** Catherine M Sweeney-Reed, Doreen Wolff, Jakob Niggel, Michael Kabesch, Christian Apfelbacher

**Affiliations:** 1 Neurocybernetics and Rehabilitation Department of Neurology Otto von Guericke University Magdeburg Magdeburg Germany; 2 Center for Behavioral Brain Sciences Otto von Guericke University Magdeburg Magdeburg Germany; 3 Institute of Social Medicine and Health Systems Research Otto von Guericke University Magdeburg Magdeburg Germany; 4 University of Regensburg Regensburg Germany; 5 MaganaMed GbmH Regensburg Germany; 6 University Children's Hospital Regensburg (KUNO) Hospital St. Hedwig of the Order of St. John Regensburg Germany; 7 Research and Development Campus Regensburg (WECARE) Hospital St. Hedwig of the Order of St. John and University of Regensburg Regensburg Germany

**Keywords:** SARS-CoV-2, COVID-19, schools, pool testing, gargle test, test strategy, monitoring, surveillance, PCR

## Abstract

**Background:**

School closures are a widely implemented strategy for limiting infection spread in the current COVID-19 pandemic. The negative impact of school closures on children and young people is increasingly apparent, however.

**Objective:**

We aim to evaluate the feasibility of an infection monitoring program in schools to enable targeted quarantining to replace school closures. The program is currently being implemented in two model schools in Magdeburg, Germany, within the framework of the Study of Coronavirus Outbreak Prevention in Magdeburg Schools (Studie zur Ausbruchsvermeidung von Corona an Magdeburger Schulen [STACAMA]).

**Methods:**

Five pupils per class are pseudorandomly selected twice a week and asked to provide a gargle sample over a 16-week evaluation period. RNA is extracted from each sample individually in a laboratory and pooled according to school class for real-time reverse transcription polymerase chain reaction (rRT-PCR) analysis. Immediate individual sample testing will be carried out in the case of a positive pool test. Individual RNA extraction prior to pooling and application of rRT-PCR result in high test sensitivity. Testing will be performed in strict adherence to data protection standards. All participating pupils will receive a 16-digit study code, which they will be able to use to access their test

**Results:**

When the study commenced on December 2, 2020, 520 (52%) pupils and their families or guardians had consented to study participation. The study was suspended after four test rounds due to renewed school closures resulting from rising regional infection incidence. Testing resumed when schools reopened on March 8, 2021, at which time consent to participation was provided for 54% of pupils. We will quantitatively and qualitatively evaluate the logistics and acceptability of the program.

**Conclusions:**

The findings from this study should inform the design of infection surveillance programs in schools based on gargle samples and a PCR-based pool testing procedure, enabling the identification of aspects that may require adaptation before large-scale implementation. Our focus on each step of the logistics and on the experiences of families should enable a robust assessment of the feasibility of such an approach.

**International Registered Report Identifier (IRRID):**

DERR1-10.2196/28673

## Introduction

### Background

The COVID-19 pandemic declared in March 2020 by the World Health Organization, caused by the spread of the novel coronavirus SARS-CoV-2, has resulted in unforeseen challenges to education systems around the world. Although school closures have been a widely implemented measure to limit viral transmission, closing educational establishments has resulted in a plethora of adverse consequences, many of which had not been considered at the outset, for the current generation of children and young people. In addition to the negative impact on educational attainment, a myriad of further repercussions is coming to light, including detrimental effects on general health, social development, and mental well-being [1-4]. Wide-ranging sequelae have included failure to detect cases of child abuse [5-8], as well as obesity [9], and also undernourishment among children usually reliant on school lunches [10]. The impact has been greatest on children in socially disadvantaged circumstances [11,12], especially children of primary school age [13]. School closures can also play an important role in health care planning when essential clinical staff have children of school age [14]. When emergency care cannot be provided or is unsuitable due to the child belonging to a risk group, grandparents may be called upon to take on a care role [15], despite strong evidence that older age groups are at greater risk of severe illness from COVID-19 [16-18]. Although it was apparent from the early stages of the pandemic that children can be affected by SARS-CoV-2 [19], it is now also well established that children tend to have milder acute symptoms [20,21], and younger children have lower SARS-CoV-2 infection and transmission rates than adults [22-27]. Against this backdrop, the development of concepts to enable safe reopening of schools is imperative, not only during the current pandemic but also to avoid a repeat of widespread school closures in the future. Indeed, with the rising number of infections in the current pandemic resulting from new mutations of SARS-CoV-2 with higher transmission rates (variants of concern), we are already potentially facing the next wave in the current pandemic.

Despite the protective measures implemented in schools when they were reopened in the summer of 2020 following the first lockdown, including the wearing of face masks to cover nose and mouth, regular handwashing, and social distancing, the infection incidence rose, and further school closures ensued in many countries. Regular testing of the population for infection with SARS-CoV-2 was proposed early in the pandemic [28,29], and surveillance programs with contact tracing and quarantining measures have been recommended as a potential strategy to enable schools to reopen [26,30-32].

Until now, we are aware of two studies successfully implementing a regular surveillance program for the monitoring of SARS-CoV-2 infections in schools, both of which involve collection of swabs by health care staff for real-time reverse transcription polymerase chain reaction (rRT-PCR) testing [33,34]. Despite massive expansion of testing capacities, however, regular testing and provision of rapid results for all school children would place a substantial logistical burden on schools. Here, we present a study protocol for the evaluation of the feasibility of an efficient SARS-CoV-2 testing strategy based on pool testing in two model schools in Magdeburg, Germany—Study of Coronavirus Outbreak Prevention in Magdeburg Schools (Studie zur Ausbruchsvermeidung von Corona an Magdeburger Schulen [STACAMA]). We aim to assess both the practical implementation of the testing procedure as well as its acceptance among pupils and their parents or guardians and teachers in a primary and a secondary school. The evaluation of the test procedure will be performed in close collaboration with the University Children’s Hospital Regensburg and Hospital St. Hedwig of the Order of St. John, Regensburg, Germany, where a similar concept is being applied in a choir-based boarding school—Study of Coronavirus Outbreak Prevention in the Cathedral Choir School (Studie zur Ausbruchsvermeidung von Corona bei den Domspatzen [STACADO]), the Regensburger Domspatzen.

### Testing Strategy

We selected a test procedure involving pool testing of RNA extracted from individual gargle samples on the basis of the noninvasiveness, ease of implementation, and cost-effectiveness of the approach. Other test procedures that may be used to detect SARS-CoV-2 infection include taking a swab from the nose, throat, and/or mouth, saliva sampling, and serology. Although a deep nasopharyngeal swab has been deemed the gold standard in testing for SARS-CoV-2, it may be considered invasive, potentially finding low acceptance among asymptomatic children as a means of regular testing. Moreover, comparable sensitivity has been demonstrated between deep nasopharyngeal swabs and the use of saliva as a test material, both among symptomatic and asymptomatic individuals [35-37]. Cost-effectiveness is an important consideration for an ongoing program. Gargling a saline solution or filtered water can be carried out independently [38,39], saving the costs of support from clinical personnel and the accompanying personal protective equipment [39]. Moreover, gargle tests have been established as providing an effective approach to diagnosing respiratory infection among children [40], and they have been shown specifically to be effective in the diagnosis of SARS-CoV-2 infection [41]. The approach is particularly well-suited to a regular monitoring program, because samples from several participants can be tested together in a so-called pool testing procedure [41-44], with lower costs than individual testing [44]. The possibility has been raised that pooling material prior to testing could potentially result in a reduced test sensitivity [44]. However, a modeling study, in which various parameters involved in potential monitoring programs were evaluated, while taking account of the dynamics of the viral load over the course of SARS-CoV-2 infection, has suggested that the frequency of testing and rapidity of provision of test results have a greater impact on case detection rates than the sensitivity of the testing method used [45].

### Aims

We aim to evaluate the implementation of a program to provide regular monitoring of SARS-CoV-2 infection occurrences among asymptomatic pupils, in order to avoid infection outbreaks and consequent school closures, based on gargle samples and a pool testing procedure. Central to the study are the evaluation of the logistics and the acceptability of the testing strategy. A further key consideration in the study design was the rapid communication of test results in accordance with national data protection standards.

## Methods

### Overview of Study Design

Asymptomatic pupils attending a primary and a secondary school in Magdeburg, Germany are being monitored for infection with SARS-CoV-2 over a period of 16 study weeks. The study commenced on December 2, 2020. Twice a week, five pupils are pseudorandomly selected from each class and invited to provide a gargle sample for analysis in a pool test procedure, resulting in up to 8 pools from the primary school and 26 pools from the secondary school. A positive pool test will be followed immediately by testing of the individual samples from that pool. After 3 weeks of testing, a questionnaire regarding the reasons behind the choice over whether to participate was distributed to all families. Further questionnaires among families and teachers, focusing on the acceptance of the test strategy, are planned for halfway through and on completion of the study. In the case of further lockdowns, the program will be, as was until recently the case, temporarily suspended, and then resumed when schools reopen.

### Study Population

#### Recruitment

The study was presented at school parent evenings by a member of the study team (CMSR), the heads of the schools, and at the primary school, also by University Hospital Magdeburg management. The evenings were attended by up to two elected parent representatives per class. An overview of the planned study invitation was given during separate parent evenings for each individual class by the class representatives, aided by a written summary of the study information. Subsequently, the complete study information, as approved by the Local Ethics Commission of the University Hospital Magdeburg, was distributed to all families, both in paper form and electronically, by email, and through the study website (STACAMA Homepage [46]). The formal invitation encompassed the following study documents: study information sheet, data protection declaration, consent form, as well as a flyer explaining how to provide a gargle sample using the solution provided. The documents were made available in additional languages, through professional translation, as required. Following evaluation of the response rates, reminder letters were forwarded via the heads of the schools and parent representatives. A dedicated study website is available and will be maintained throughout the study period, with an email contact and a study telephone hotline (DW) provided for any questions.

#### Inclusion and Exclusion Criteria

The study population includes pupils aged between 6 and 18 years (primary school: age range 6-10 years, 2 classes per grade, 20-24 pupils per class; secondary school: age range 10-18 years, 4 classes per grade in grades 5-10, 22-30 pupils per class, and 93 and 96 pupils in grades 11 and 12 respectively, with mixing of groups according to course selection). Inclusion in the study requires provision of written consent from the parents or guardians, as well as written consent from the pupil, depending on age.

School attendance was dependent on pupils being asymptomatic and having had no contact with persons confirmed to be infected with SARS-CoV-2 in the preceding 14 days. Parents or guardians were required by the schools to provide weekly written confirmation of these statements before the school closures in December 2020.

A minimum participation rate of 60% per class was deemed necessary, because our aim is to assess the feasibility of a surveillance program based on pseudorandom sampling to monitor for SARS-CoV-2 infections in an asymptomatic cohort. A lower participation rate would change the program to a regular testing of the same individuals. The evaluation of the burden of more frequent testing would require a separate study. Pupils may be included at a later date, following study commencement, should they so choose, up until the end of the study period, potentially enabling further classes to reach the 60% participation level required for testing to commence.

#### Termination of Participation

Study participants can terminate their participation at any time, without providing reasons. If a participant leaves the study, the test results will be retained, in a completely anonymized form. Study personnel are not able to link the data with any individual person. All information is saved in the study app under a 16-digit code, which is known only to the participant. Deletion of individual data is possible, but only if the participant discloses their 16-digit code.

#### Ethics and Consent

The study protocol was developed to meet the standards and gain approval from the Coordination Center for Clinical Studies, Magdeburg, and the Data Protection Advisory Service of the University Hospital Magdeburg. The Local Ethics Committee of the Otto von Guericke University Magdeburg has evaluated the STACAMA study and agreed to its implementation (164/2). Participation in the study is voluntary. Participants are informed about the goals and content of the study, as well as over the data protection, and written, informed consent is a prerequisite for inclusion in the study.

#### Data Protection

The study is being carried out under strict adherence to data protection standards set out by the General Data Protection Regulation in the European Union (EU) (EU-Datenschutzgrundverordnung [DSGVO]) and the Federal Data Protection Act (the German Bundesdatenschutzgesetzes [BDSG]). All data are secured against unauthorized access and are stored on a secure server in Frankfurt, Germany, whose infrastructure is certified as being in accordance with ISO 27001 standards. All aspects of data transfer are encrypted, in accordance with the recommendations set out under the AES-256 standard. Anonymized data may be provided to academic third parties for analysis and evaluation of academic and medical hypotheses. The data will not be used for purposes beyond the aims of this study or, in particular, for commercial purposes. After the end of the study evaluation, the data will be deleted, at the latest on December 31, 2021.

### Study Procedures

#### Hygiene Measures

Before commencement of the study, each school head developed a hygiene policy suited to the relevant age groups and school facilities, in collaboration with the Department of Hygiene at the University Hospital Magdeburg and the study team (CMSR). At the beginning of the school year, an educational project week was provided at the primary school by members of the Departments of Hygiene, Management, and Public Outreach at the University Hospital Magdeburg and the study team (CMSR), in which SARS-CoV-2 and the hygiene policy were introduced and explained using an interactive, age-relevant approach for each year group. At the secondary school, members of the Department of Hygiene held a series of presentations regarding hygiene and the limitation of the spread of SARS-CoV-2 for all pupils, for two consecutive year groups at a time to enable an age-appropriate delivery as well as compliance with social distancing requirements, with an opportunity to ask questions.

The hygiene policies at both schools involved strict separation of classes into separate cohorts. Wearing of face masks covering the nose and mouth was mandatory in the secondary school at all times, including when the pupils were outside. In the primary school, face masks were also mandatory, except during lessons and while on the playground outside. Classrooms were required to be aired for a minimum of 5 minutes at least once per 45-minute lesson in the primary school and at least twice per 45-minute lesson in the secondary school. Hand hygiene was emphasized in both schools, and running water, soap, and disposable paper hand towels were provided in every classroom. Disinfectant dispensers were available at the school and cafeteria entrances in the secondary school, which could be operated with the forearm, and pupils were asked to bring disinfectants for personal use. School desks were rearranged to provide maximum distance between pupils. The school corridors in both schools were signposted to separate direction of walking, and a minimal distance of 1.5 meters between individuals was emphasized. One-way systems were implemented in corridors and on staircases where possible. In the primary school only, sport lessons were carried out outside and with distancing measures, and singing was only permitted on an individual basis, with pupils maintaining a minimum distance of 2 meters from each other. Sport and singing were not permitted at the secondary school, and school lunches were provided in small groups and no longer in a buffet format.

#### Testing

**Figure 1:**
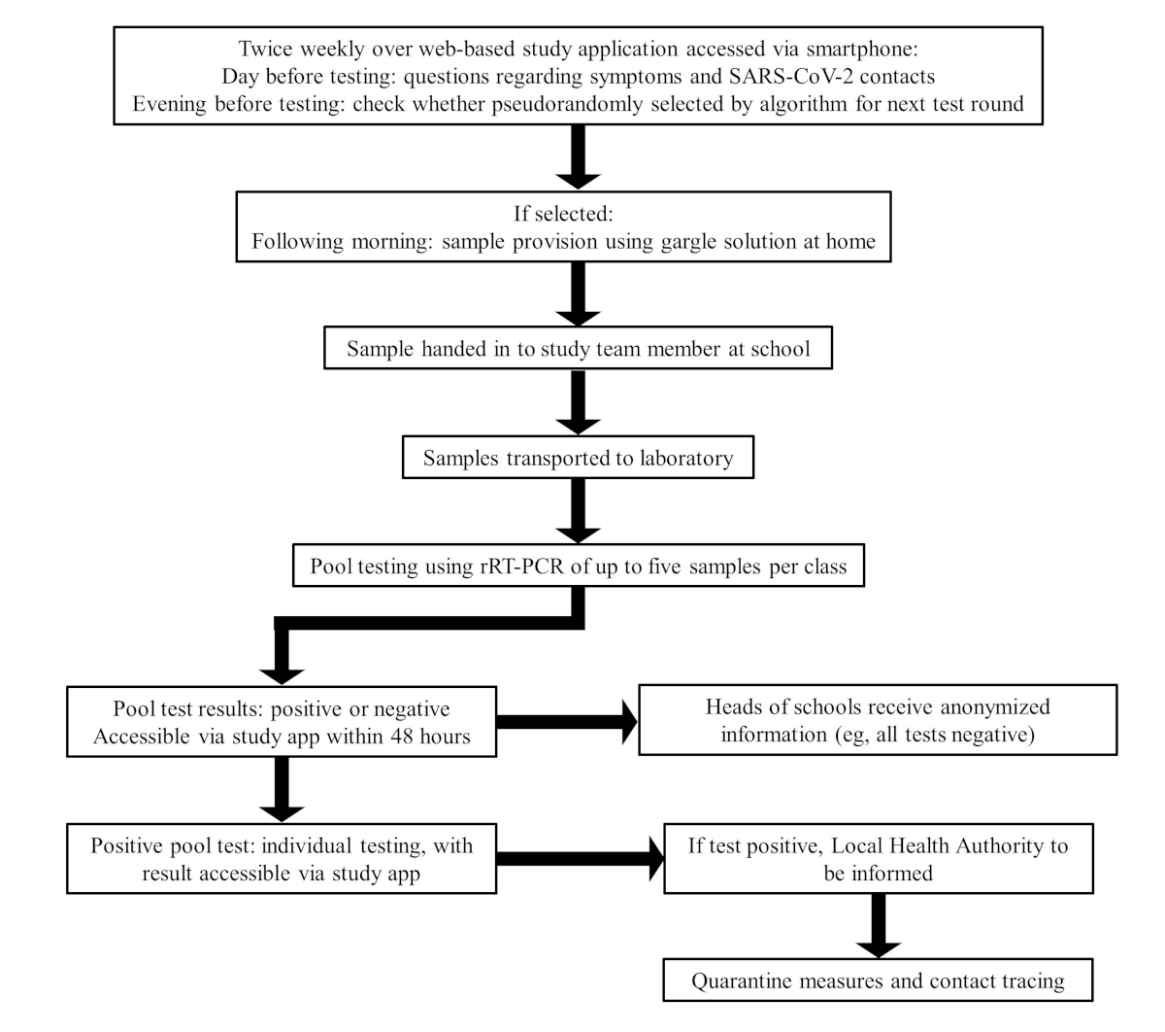
Monitoring procedure based on gargle samples and pool testing. rRT-PCR: real-time reverse transcription polymerase chain reaction.

The surveillance program is performed as illustrated in Figure 1. On enrollment in the study, each pupil is given a test kit for provision of a gargle sample, accompanied by an instruction leaflet and an information sheet with a unique 16-digit participant code and instructions on how to use the study app, which is implemented on the qnome platform [47]. The study data are recorded and stored under this unique code. The assignment of the access codes to individual participants is not recorded and stored, so that it is not possible to associate participant data with individuals.

Pupils or their parents or guardians are asked to enter their 16-digit access code into the study app each Sunday and Tuesday evening to ascertain whether they have been pseudorandomly selected through the study app to provide a gargle sample for inclusion in the test pool for their class the following morning (Mondays and Wednesdays). On entering the code for the first time, participants are asked to complete a voluntary starting questionnaire, in which they are asked whether a member of the household is employed in health or social services. Before each test session, a further questionnaire is completed within the study app, in which participants are asked whether a family member or friend has tested positive for SARS-CoV-2 since the previous questionnaire and about relevant symptoms. An individual risk profile is then generated according to a point allocation system, and participants are selected for the next testing session through an algorithm with weighting towards selection of participants at increased risk of infection.

The samples are collected independently at home in order to avoid the risk of spreading infection through gargling on the school premises. Participants are asked to provide a sample through deep throat gargling with 10 mL of 0.9% NaCl solution. This sample collection procedure does not involve a health risk to the participants. After gargling, the solution is collected in a container and the lid is screwed shut. A sample tube containing a vacuum is also provided, which is appended to an opening in the lid of the container. The gargle sample is then evacuated into the sealed sample tube. The sample tube has a laboratory-generated barcode, which is scanned using a smartphone camera linked to the study app. The participant’s 16-digit personal code is thus linked with the sample, which allows the participant to access their test results subsequently using the app. The frequency with which a pupil is tested can also be determined via this code, without compromising anonymity. Participants are also requested to write their first and last names and class on the sample tube. This information is a legal requirement, as infection with SARS-CoV-2 is notifiable. The processing laboratory is required to provide personal identification information to the Local Health Authority to enable quarantine measures to be applied and contact tracing to be carried out. The school is subsequently legally required to provide the relevant individual contact information to the Local Health Authority. This procedure is in place independently from the study and is implemented when SARS-CoV-2 infection is confirmed in any person attending the school, regardless of where the testing was performed.

After samples are transported to the laboratory by an independent contractor, pooling of the individually extracted RNA takes place on the basis of class attendance, because hygiene measures in place in both participating schools ensure that pupils in the same class form a consistent cohort. The pool is then tested using a rRT-PCR–based direct detection of SARS-CoV-2. In the case of a positive pool test result, the samples involved in the pooling procedure will be immediately tested individually and the results, as a yes/no response, will be assigned to the 16-digit access code. The test results will be visible via the study app to the respective pupils and their parents or guardians within 48 hours. The pool testing procedure was developed by the participating commercial laboratory and is currently being registered for patenting. Internal evaluation procedures have thus far revealed no loss of sensitivity in comparison with single specimen measurements.

The measures to be implemented in the case of a positive test result were agreed in advance with the Local Health Authority responsible for imposing protective measures and contact tracing for notifiable diseases. The results from the previous two testing rounds are additionally available, to enable early, rapid recognition of an outbreak. Pupils identified as contacts of a person infected with SARS-CoV-2 will be informed by the Local Health Authority. Whether the identification of a positive test for SARS-CoV-2 infection in a school results in quarantine measures for the entire class, course group, or similar, in addition to close contacts, lies at the discretion of the responsible Local Health Authority and is continually updated on the basis of local and national rates of infection. The recommendations of the Robert Koch Institute may be adapted to the local situation in accordance with the intended protection goals.

#### Evaluation Questionnaire

To evaluate the acceptance of the test strategy among pupils and their families, a questionnaire was provided 3 weeks after study commencement, in paper and electronic formats, to the families of all pupils. The questionnaire could be voluntarily and anonymously completed, regarding the reasons behind decisions over whether to participate. Halfway through and at the end of the 16 testing weeks, families and teachers will be asked about their experiences.

### Data Analysis Plan and Endpoints

This study has two primary endpoints: the evaluation of the logistical implementation of the surveillance program and of its acceptance among participants and their families and teachers. The logistical implementation will be assessed according to the number of pool tests successfully performed per participating class. The stages in the procedure will be evaluated individually, including the following: (1) number of pretest web-based questionnaires completed; (2) number of samples handed in at the schools; (3) arrival of samples at the laboratory within the recommended time frame for sample transportation; (4) analysis of pools and when a pool test is positive, individual samples; and (5) provision of results accessible via the study app within 48 hours. The acceptance will be evaluated in the following four ways: (1) the participant quota as a whole, for each school, and for each year group; (2) participation of additional year groups over the study period; (3) the study dropout rate; and (4) evaluation of the questionnaires.

## Results

The recruitment of participants for the STACAMA study began when the 2020/21 school year commenced, and inclusion will continue to be possible until the end of the study period. Study information was distributed to 1003 pupils, and 520 (52%) pupils and/or their parents or guardians had provided written consent to participation by the time testing began. The 60% participation rate required for testing commencement was reached, when the study commenced on December 2, 2020, in the third (62%), fourth (61%), and fifth (70%) grades. The study was suspended on December 15, 2020, after four testing rounds, due to renewed school closures. During that time period, no pool tests were positive. Subsequent rounds of testing commenced on March 8, 2021, when the schools reopened, and will continue until 16 study weeks have been completed in total. The 60% inclusion rate has now also been met in additional classes, which will now be included: two classes in the sixth grade (66%), one in the seventh grade (76%), and two in the eighth grade (62%). Consent to participation has increased to 63% in the third grade and 73% in the fifth grade. Across all grades, consent to participation is currently 54%. Study results will be published in peer-reviewed scientific journals.

## Discussion

This study will provide insights into the feasibility of a surveillance program in schools for the prevention of SARS-CoV-2 outbreaks through regular gargle sampling and pool testing of randomly selected asymptomatic pupils. Such a program has the potential to enable schools to reopen safely, avoiding the far-reaching negative consequences of school closures. Through evaluating each stage of the proposed testing strategy, we expect to be able to establish which steps in the program can be successfully implemented, while identifying aspects that could require alternative solutions. The acceptability of such a program is paramount if it is to be applied on a regular basis in all schools. The open questions in our questionnaire should allow us to gain an understanding of the impact of infection monitoring on pupils as well as their families and teachers. The use of gargle tests offers potential advantages over surveillance programs based on swab testing. Sample collection is noninvasive, safe, and can be performed independently at home, without support from medical personnel and the requirement of personal protective equipment or a risk of infection spread during sample collection in schools. Furthermore, the samples can be readily used in a pool testing procedure. Pool testing offers a potentially efficient approach to monitoring infection in a low-prevalence environment, which is an important consideration given finite laboratory capacity and the scale of testing required for all schools to be included in such a surveillance program.

## Abbreviations

BDSG: Bundesdatenschutzgesetz (Federal Data Protection Act)
DSGVO: Datenschutzgrundverordnung (General Data Protection Regulation)
EU: European Union
rRT-PCR: real-time reverse transcription polymerase chain reaction
STACAMA: Studie zur Ausbruchsvermeidung von Corona an Magdeburger Schulen (Study of Coronavirus Outbreak Prevention in Magdeburg Schools)
STACADO: Studie zur Ausbruchsvermeidung von Corona bei den Domspatzen (Study of Coronavirus Outbreak Prevention in the Cathedral Choir School)

## References

[1] Ashikkali L, Carroll W, Johnson C (2020). The indirect impact of COVID-19 on child health. Paediatr Child Health (Oxford).

[2] Giannopoulou I, Efstathiou V, Triantafyllou G, Korkoliakou P, Douzenis A (2021). Adding stress to the stressed: Senior high school students' mental health amidst the COVID-19 nationwide lockdown in Greece. Psychiatry Res.

[3] Rajabi M (2020). Mental health problems amongst school-age children and adolescents during the COVID-19 pandemic in the UK, Ireland and Iran: A call to action and research. Health Promot Perspect.

[4] Viner RM, Russell SJ, Croker H, Packer J, Ward J, Stansfield C, Mytton O, Bonell C, Booy R (2020). School closure and management practices during coronavirus outbreaks including COVID-19: a rapid systematic review. Lancet Child Adolesc Health.

[5] Baron E, Goldstein E, Wallace C (2020). Suffering in silence: How COVID-19 school closures inhibit the reporting of child maltreatment. J Public Econ.

[6] Crawley E, Loades M, Feder G, Logan S, Redwood S, Macleod J (2020). Wider collateral damage to children in the UK because of the social distancing measures designed to reduce the impact of COVID-19 in adults. BMJ Paediatr Open.

[7] Garstang J, Debelle G, Anand I, Armstrong J, Botcher E, Chaplin H, Hallett N, Morgans C, Price M, Tan EEH, Tudor E, Taylor J (2020). Effect of COVID-19 lockdown on child protection medical assessments: a retrospective observational study in Birmingham, UK. BMJ Open.

[8] Khan T, Mikuska É (2021). The first three weeks of lockdown in England: The challenges of detecting safeguarding issues amid nursery and primary school closures due to COVID-19. Social Sciences & Humanities Open.

[9] Rundle AG, Park Y, Herbstman JB, Kinsey EW, Wang YC (2020). COVID-19-related school closings and risk of weight gain among children. Obesity (Silver Spring).

[10] Mayurasakorn K, Pinsawas B, Mongkolsucharitkul P, Sranacharoenpong K, Damapong S (2020). School closure, COVID-19 and lunch programme: Unprecedented undernutrition crisis in low-middle income countries. J Paediatr Child Health.

[11] Van Lancker W, Parolin Z (2020). COVID-19, school closures, and child poverty: a social crisis in the making. Lancet Public Health.

[12] Viner RM, Bonell C, Drake L, Jourdan D, Davies N, Baltag V, Jerrim J, Proimos J, Darzi A (2021). Reopening schools during the COVID-19 pandemic: governments must balance the uncertainty and risks of reopening schools against the clear harms associated with prolonged closure. Arch Dis Child.

[13] Tomasik M, Helbling L, Moser U Educational gains of in-person vs. distance learning in primary and secondary schools: A natural experiment during the COVID-19 pandemic school closures in Switzerland. Int J Psychol..

[14] Bayham J, Fenichel EP (2020). Impact of school closures for COVID-19 on the US health-care workforce and net mortality: a modelling study. Lancet Public Health.

[15] Feng Z, Hill AN, Curns AT, Glasser JW (2017). Evaluating targeted interventions via meta-population models with multi-level mixing. Math Biosci.

[16] Grasselli G, Zangrillo A, Zanella A, Antonelli M, Cabrini L, Castelli A, Cereda D, Coluccello A, Foti G, Fumagalli R, Iotti G, Latronico N, Lorini L, Merler S, Natalini G, Piatti A, Ranieri MV, Scandroglio AM, Storti E, Cecconi M, Pesenti A, COVID-19 Lombardy ICU Network (2020). Baseline characteristics and outcomes of 1591 patients infected with SARS-CoV-2 admitted to ICUs of the Lombardy region, Italy. JAMA.

[17] Sun K, Chen J, Viboud C (2020). Early epidemiological analysis of the coronavirus disease 2019 outbreak based on crowdsourced data: a population-level observational study. Lancet Digit Health.

[18] Wu Z, McGoogan JM (2020). Characteristics of and important lessons from the coronavirus disease 2019 (COVID-19) outbreak in China: Summary of a report of 72 314 cases from the Chinese Center for Disease Control and Prevention. JAMA.

[19] Dong Y, Mo X, Hu Y, Qi X, Jiang F, Jiang Z, Tong S (2020). Epidemiology of COVID-19 among children in China. Pediatrics.

[20] Davies NG, Klepac P, Liu Y, Prem K, Jit M, Eggo RM, CMMID COVID-19 working group (2020). Age-dependent effects in the transmission and control of COVID-19 epidemics. Nat Med.

[21] Ludvigsson JF (2020). Systematic review of COVID-19 in children shows milder cases and a better prognosis than adults. Acta Paediatr.

[22] Ehrhardt J, Ekinci A, Krehl H, Meincke M, Finci I, Klein J, Geisel B, Wagner-Wiening C, Eichner M, Brockmann SO (2020). Transmission of SARS-CoV-2 in children aged 0 to 19 years in childcare facilities and schools after their reopening in May 2020, Baden-Württemberg, Germany. Euro Surveill.

[23] Heavey L, Casey G, Kelly C, Kelly D, McDarby G (2020). No evidence of secondary transmission of COVID-19 from children attending school in Ireland, 2020. Euro Surveill.

[24] Munro APS, Faust SN (2020). Children are not COVID-19 super spreaders: time to go back to school. Arch Dis Child.

[25] Rajmil L (2020). Role of children in the transmission of the COVID-19 pandemic: a rapid scoping review. BMJ Paediatr Open.

[26] Viner RM, Mytton OT, Bonell C, Melendez-Torres GJ, Ward J, Hudson L, Waddington C, Thomas J, Russell S, van der Klis F, Koirala A, Ladhani S, Panovska-Griffiths J, Davies NG, Booy R, Eggo RM (2021). Susceptibility to SARS-CoV-2 infection among children and adolescents compared with adults: A systematic review and meta-analysis. JAMA Pediatr.

[27] Buchholz U, Lehfeld A, Otte IKE, Lindahl M, Lewandowsky M, Hauer B, Pozo MF, el BC, Hanefeld J, Haas W (2021). Epidemiology of COVID-19 in a school setting. Article in German. Epid Bull 2021;13?.

[28] Peto J (2020). Covid-19 mass testing facilities could end the epidemic rapidly. BMJ.

[29] Peto J, Alwan NA, Godfrey KM, Burgess RA, Hunter DJ, Riboli E, Romer P, 27 signatories (2020). Universal weekly testing as the UK COVID-19 lockdown exit strategy. Lancet.

[30] Otte Im Kampe E, Lehfeld A, Buda S, Buchholz U, Haas W (2020). Surveillance of COVID-19 school outbreaks, Germany, March to August 2020. Euro Surveill.

[31] Walger P, Heininger U, Knuf M, Exner M, Popp W, Fischbach T, Trapp S, Hübner J, Herr C, Simon A, German Society for Hospital Hygiene (DGKH), German Society for Pediatric Infectious Diseases (DGPI), German Academy for PediatricAdolescent Medicine (DAKJ), Society of Hygiene‚ EnvironmentalPublic Health Sciences (GHUP), Professional Association of Pediatricians in Germany (bvkj e.V.) (2020). Children and adolescents in the CoVid-19 pandemic: Schools and daycare centers are to be opened again without restrictions. The protection of teachers, educators, carers and parents and the general hygiene rules do not conflict with this. GMS Hyg Infect Control.

[32] Ziauddeen N, Woods-Townsend K, Saxena S, Gilbert R, Alwan N (2020). Schools and COVID-19: Reopening Pandora’s box?. Public Health in Practice.

[33] Hoch M, Vogel S, Kolberg L, Dick E, Fingerle V, Eberle U, Ackermann N, Sing A, Huebner J, Rack-Hoch A, Schober T, von BU Weekly SARS-CoV-2 sentinel in primary schools, kindergartens and nurseries, June to November 2020, Germany. medRxiv..

[34] Kriemler S, Ulyte A, Ammann P, Peralta G, Berger C, Puhan M, Radtke T Surveillance of acute SARS-CoV-2 infections in school children and point-prevalence during a time of high community transmission in Switzerland. medRxiv..

[35] Kojima N, Turner F, Slepnev V, Bacelar A, Deming L, Kodeboyina S, Klausner J (2020). Self-collected oral fluid and nasal awab specimens demonstrate comparable sensitivity to clinician-collected nasopharyngeal swab specimens for the detection of SARS-CoV-2. Clin Infect Dis.

[36] Procop GW, Shrestha NK, Vogel S, Van Sickle K, Harrington S, Rhoads DD, Rubin BP, Terpeluk P (2020). A direct comparison of enhanced saliva to nasopharyngeal swab for the detection of SARS-CoV-2 in symptomatic patients. J Clin Microbiol.

[37] Schwob J, Miauton A, Petrovic D, Perdrix J, Senn N, Jaton K, Onya O, Maillard A, Minghelli G, Cornuz J, Greub G, Genton B, D'Acremont V Antigen rapid tests, nasopharyngeal PCR and saliva PCR to detect SARS-CoV-2: a prospective comparative clinical trial. medRxiv..

[38] Goldfarb D, Tilley P, Al-Rawahi G, Srigley J, Ford G, Pedersen H, Pabbi A, Hannam-Clark S, Charles M, Dittrick M, Gadkar V, Pernica J, Hoang L (2021). Self-collected saline gargle samples as an alternative to healthcare worker collected nasopharyngeal swabs for COVID-19 diagnosis in outpatients. J Clin Microbiol.

[39] Malecki M, Lüsebrink Jessica, Teves S, Wendel AF (2021). Pharynx gargle samples are suitable for SARS-CoV-2 diagnostic use and save personal protective equipment and swabs. Infect Control Hosp Epidemiol.

[40] Morikawa S, Hiroi S, Kase T (2015). Detection of respiratory viruses in gargle specimens of healthy children. J Clin Virol.

[41] Ibette LG, de CCCR, Oliveira SV, Mayumi AC, Spinazola DPL, Pereira DSF, Bernardes BDSD, Corrêa DOSK, Aparecida BM, Nery RW, Cilli A, Monteiro ME, Maria SAA, do CSTTM, Fernando DMBL, Fernando DMBPL SARS-CoV-2 RNA detection using pooling of self-collected samples: Simple protocol may foster asymptomatic surveillance. medRxiv..

[42] Yelin I, Aharony N, Tamar ES, Argoetti A, Messer E, Berenbaum D, Shafran E, Kuzli A, Gandali N, Shkedi O, Hashimshony T, Mandel-Gutfreund Y, Halberthal M, Geffen Y, Szwarcwort-Cohen M, Kishony R (2020). Evaluation of COVID-19RT-qPCR test in multi-sample pools. Clin Infect Dis.

[43] Abdalhamid B, Bilder C, McCutchen E, Hinrichs S, Koepsell S, Iwen P (2020). Assessment of specimen pooling to conserve SARS CoV-2 testing resources. Am J Clin Pathol.

[44] Singh AK, Nema RK, Joshi A, Shankar P, Nema S, Raghuwanshi A, Patankar C, Mathew BJ, Shrivas A, Pandey R, Tripathi R, Biswas D, Singh S (2020). Evaluation of pooled sample analysis strategy in expediting case detection in areas with emerging outbreaks of COVID-19: A pilot study. PLoS One.

[45] Larremore DB, Wilder B, Lester E, Shehata S, Burke JM, Hay JA, Tambe M, Mina MJ, Parker R (2021). Test sensitivity is secondary to frequency and turnaround time for COVID-19 screening. Sci Adv.

[46] Study of Coronavirus Outbreak Prevention in Magdeburg Schools (STACAMA). Webpage in German.

[47] Qnome.

